# Supplementary material for: Prior Exposure and Toddlers’ Sleep-Related Memory for Novel Words
Source: Brain Sci. 2021 Oct 18;11(10):1366. doi: 10.3390/brainsci11101366 (PMC8534215; doi:10.3390/brainsci11101366)

Table of Contents – FULL LIST OF MODELS AND COMPARISON BETWEEN MODELS

Table S1 2

Ostensive Naming Group's One-sample t-tests Comparing Fast Mapping and Retention to Chance 2

Table S2 3

Ostensive Naming Group's Retention Across Immediate, Afternoon, and Post-nocturnal Tests 3

Table S3 3

Ostensive Naming Group's Condition by Testing Session Fixed Effects Parameter Estimates 3

Table S4 4

Comparison of Ostensive and Non-ostensive Naming Groups' Retention Across Immediate, Afternoon, Post-nocturnal Tests 4

Table S5 5

Group by Condition by Test Session Fixed Effects Parameter Estimates 5

Table S6 6

Time Intervals from Fast Mapping to Sleep Onset and the Afternoon and Post-nocturnal Tests 6

Figure S1 7

Ostensive Naming Group's Heirarchical Cluster Analysis Dendogram 7

Figure S2 8

Non-ostensive Naming Group's Heirarchical Cluster Analysis Dendogram 8

Table S1

Ostensive Naming Group's One-sample t-tests Comparing Fast Mapping and Retention to Chance

					95% Confidence Interval		
	t	***df***	***p***	**Mean Difference**	**Lower**	**Upper**	**Cohen's *d***
**Fast mapping compared to chance (0.33)**							
Nap	7.62	19	<.001	0.38	0.28	0.49	1.70
Wake	9.52	19	<.001	0.44	0.34	0.54	2.13
**Retention compared to chance (0.25)**							
Nap							
Immediate	1.53	19	.143	0.11	-0.04	0.27	0.34
Afternoon	3.71	19	.001	0.24	0.10	0.37	0.83
Post-nocturnal	3.94	19	<.001	0.22	0.11	0.34	0.88
Wake							
Immediate	4.07	19	<.001	0.21	0.10	0.32	0.91
Afternoon	4.72	19	<.001	0.29	0.16	0.41	1.06
Post-nocturnal	4.33	19	<.001	0.30	0.15	0.45	0.97

Table S2

Ostensive Naming Group's Retention Across Immediate, Afternoon, and Post-nocturnal Tests

**Set 1: *DV = retention at the 3 test sessions*; IVs = ostensive naming, condition (nap, wake), test session (immediate, afternoon, post-nocturnal), and covariate habitual napping**												
**Model specification**	**Model name**	**Fixed Effects**	**Fixed Effects added**	**Random Effects**		**Model fit**				**LRT Test**		
				**intercept**	**slope**	***AIC***	***BIC***	***-2LL***	***df***	**Comparison**	***df***	**X^2**
RE only	M1		-	participant	*	33.29	41.67	27.29	2			
FE	M2	condition	condition	participant		33.95	45.10	25.95	4	M2 vs M1	2	-1.34
FEs	M3	condition + test session	test session	participant		33.02	49.74	21.02	6	M3 vs M2	2	-4.93*
FEs + interaction	M4	condition + test session + condition × test session	condition × test session	participant		36.78	59.08	20.78	8	M4 vs M3	2	-0.24

*not included as models failed to converge with the addition of random slopes

Table S3

Ostensive Naming Group's Condition by Testing Session Fixed Effects Parameter Estimates

				**95% Confidence Interval**				
**Names**	**Effect**	**Estimate**	***SE***	**Lower**	**Upper**	***df***	***t***	***p***
(Intercept)	(Intercept)	0.48	0.03	0.42	0.54	40	14.93	< .001
condition	wake - nap	0.08	0.06	-0.05	0.20	40	1.17	.250
test session 1	linear	-0.00	0.04	-0.07	0.07	80	-0.00	.999
test session 2	quadratic	0.08	0.04	0.01	0.15	80	2.26	.027
condition ✻ test session 1	wake - nap ✻ linear	0.02	0.07	-0.12	0.16	80	0.24	.807
condition ✻ test session 2	wake - nap ✻ quadratic	-0.03	0.07	-0.17	0.11	80	-0.42	.673

Table S4

Comparison of Ostensive and Non-ostensive Naming Groups' Retention Across Immediate, Afternoon, Post-nocturnal Tests

**Set 2: *DV = retention at the 3 test sessions*; IVs = group (ostensive naming, non-ostensive naming), condition (nap, wake), test session (immediate, afternoon, post-nocturnal), and covariate habitual napping**												
**Model specification**	**Model name**	**Fixed Effects**	**Fixed Effects added**	**Random Effects**		**Model fit**				**LRT Test**		
				**intercept**	**slope**	***AIC***	***BIC***	***-2LL***	***df***	**Comparison**	***df***	**X^2**
FEs + interaction	M5	condition +<br>test session +<br>condition × test session +<br>group	group	participant		92.82	124.14	74.82	9	M5 vs M4	1	54.04*
FEs + interactions	M6	condition +<br>test session +<br>condition × test session +<br>group +<br>group × condition	group × condition	participant		86.46	121.26	66.46	10	M6 vs M5	1	-8.36*
FEs + interactions	M7	condition +<br>test session +<br>condition × test session +<br>group +<br>group × condition +<br>group × test session	group × test session	participant		78.97	120.73	54.96	12	M7 vs M6	2	-11.50*
FEs + interactions	M8	condition +<br>test session +<br>condition × test session +<br>group +<br>group × condition +<br>group × test session<br>group × condition × test session	group × condition × test session	participant		81.81	130.54	53.82	14	M8 vs M7	2	-1.14

Table S5

Group by Condition by Test Session Fixed Effects Parameter Estimates

				95% Confidence Interval				
**Names**	**Effect**	**Estimate**	***SE***	**Lower**	**Upper**	***df***	***t***	***p***
(Intercept)	(Intercept)	0.43	0.02	0.39	0.47	40.21	19.61	< .001
condition	wake - nap	-0.05	0.04	-0.13	0.03	125.03	-1.31	.193
test session1	linear	-0.03	0.03	-0.08	0.03	190.61	-0.95	.342
test session2	quadratic	-0.01	0.03	-0.07	0.04	190.61	-0.48	.630
group	non_ost - ost	-0.11	0.03	-0.17	-0.04	221.36	-3.12	.002
condition ✻ test session1	wake - nap ✻ linear	0.02	0.06	-0.09	0.13	190.61	0.38	.704
condition ✻ test session2	wake - nap ✻ quadratic	-0.09	0.06	-0.21	0.02	190.61	-1.61	.110
condition ✻ group	wake - nap ✻ non_ost - ost	-0.22	0.07	-0.37	-0.08	207.76	-2.99	.003
test session1 ✻ group	linear ✻ non_ost - ost	-0.05	0.06	-0.17	0.06	190.61	-0.95	.342
test session2 ✻ group	quadratic ✻ non_ost - ost	-0.19	0.06	-0.30	-0.08	190.61	-3.32	.001
condition ✻ test session1 ✻ group	wake - nap ✻ linear ✻ non_ost - ost	0.01	0.12	-0.22	0.23	190.61	0.07	.941
condition ✻ test session2 ✻ group	wake - nap ✻ quadratic ✻ non_ost - ost	-0.12	0.12	-0.35	0.10	190.61	-1.07	.284

Table S6

Time Intervals from Fast Mapping to Sleep Onset and the Afternoon and Post-nocturnal Tests

	Fast Mapping to First Sleep Onset		Fast Mapping to Afternoon Test		Fast Mapping to Post-nocturnal Test	
	M	*SD*	*M*	*SD*	*M*	*SD*
Ostensive						
Nap Condition (mins)	145.15	78.94	290.35	49.30	1304.65	53.12
(hours)	(2.42)	(1.32)	(4.84)	(0.82)	(21.74)	(0.89)
Wake Condition (mins)	439.70	170.21	220.65	49.07	1324.10	104.45
(hours)	(7.33)	(2.84)	(3.68)	(0.82)	(22.07)	(1.74)
Non-ostensive						
Nap Condition (mins)	90.40	46.48	220.80	26.69	1259.70	55.12
(hours)	(1.51)	(0.77)	(3.68)	(0.44)	(21.00)	(0.92)
Wake Condition (mins)	479.30	116.35	206.30	39.10	1294.80	48.12
(hours)	(7.99)	(1.94)	(3.44)	(0.65)	(21.58)	(0.80)

Figure S1

Ostensive Naming Group's Hierarchical Cluster Analysis Dendrogram

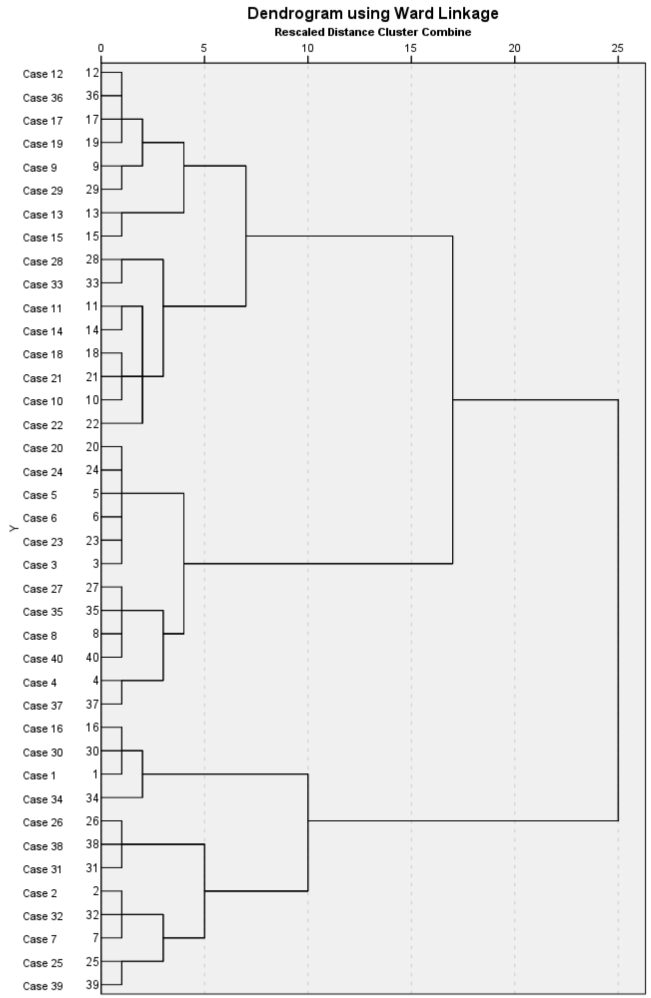

Figure S2

Non-ostensive Naming Group's Hierarchical Cluster Analysis Dendrogram

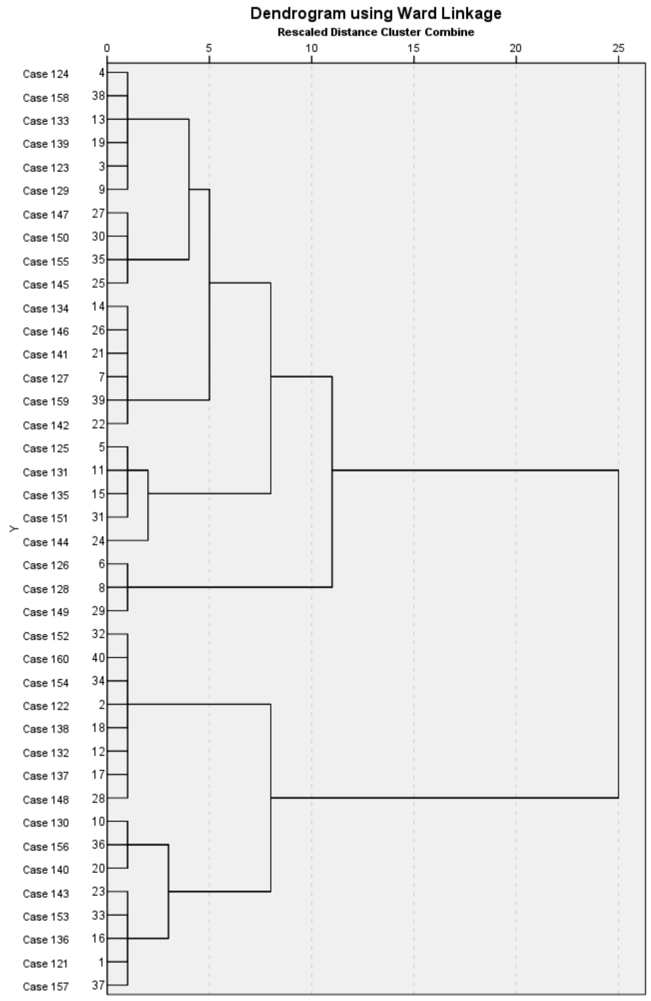

Supplement: Supplementary file 1 [file brainsci-11-01366-s001.zip › brainsci-1389456-supplementary.pdf]
